# The association of Alu repeats with the generation of potential AU-rich elements (ARE) at 3' untranslated regions.

**DOI:** 10.1186/1471-2164-5-97

**Published:** 2004-12-21

**Authors:** Hyeong Jun An, Doheon Lee, Kwang Hyung Lee, Jonghwa Bhak

**Affiliations:** 1 BioSystems Dept., Korea Advanced Institute of Science and Technology (KAIST) 373-1 Guseong-dong, Yuseong-gu, Daejeon 305-701, Republic of Korea; 2 NGIC, KRIBB, Daejeon, Korea; 3 BiO institute, Daejeon, Korea; 4 OITEK (Inc), Daejeon, Korea

## Abstract

**Background:**

A significant portion (about 8% in the human genome) of mammalian mRNA sequences contains AU (Adenine and Uracil) rich elements or AREs at their 3' untranslated regions (UTR). These mRNA sequences are usually stable. However, an increasing number of observations have been made of unstable species, possibly depending on certain elements such as Alu repeats. ARE motifs are repeats of the tetramer AUUU and a monomer A at the end of the repeats ((AUUU)_n_A). The importance of AREs in biology is that they make certain mRNA unstable. Proto-oncogene, such as c-fos, c-myc, and c-jun in humans, are associated with AREs. Although it has been known that the increased number of ARE motifs caused the decrease of the half-life of mRNA containing ARE repeats, the exact mechanism is as of yet unknown. We analyzed the occurrences of AREs and Alu and propose a possible mechanism for how human mRNA could acquire and keep AREs at its 3' UTR originating from Alu repeats.

**Results:**

Interspersed in the human genome, Alu repeats occupy 5% of the 3' UTR of mRNA sequences. Alu has poly-adenine (poly-A) regions at its end, which lead to poly-thymine (poly-T) regions at the end of its complementary Alu. It has been found that AREs are present at the poly-T regions. From the 3' UTR of the NCBI's reference mRNA sequence database, we found nearly 40% (38.5%) of ARE (Class I) were associated with Alu sequences (Table 1) within one mismatch allowance in ARE sequences. Other ARE classes had statistically significant associations as well. This is far from a random occurrence given their limited quantity. At each ARE class, random distribution was simulated 1,000 times, and it was shown that there is a special relationship between ARE patterns and the Alu repeats.

**Conclusion:**

AREs are mediating sequence elements affecting the stabilization or degradation of mRNA at the 3' untranslated regions. However, AREs' mechanism and origins are unknown. We report that Alu is a source of ARE. We found that half of the longest AREs were derived from the poly-T regions of the complementary Alu.

**Table 1:** Defined ARE classes. (Symbol marks are used in this study instead of full sequences.)

	Symbol	ARE sequence
Class I	(AUUU)5A	AUUUAUUUAUUUAUUUAUUUA
Class II	(AUUU)4A	AUUUAUUUAUUUAUUUA
Class III	U(AUUU)3AU	UAUUUAUUUAUUUAU
Class IV	UU(AUUU)2AUU	UUAUUUAUUUAUU
Class V	U4AUUUAU4	UUUUAUUUAUUUU
Class VI	W3UAUUUAUW3	WWWUAUUUAWWW

## Background

Varying more than ten-fold, messenger RNA degradation is essential for the regulation of gene expression [1,2]. Differential mRNA decay rates were determined by specific *cis*-acting sequences within mRNA. For example, the mRNA sequences of yeast, many mammalians, and other eukaryotes contain AU-rich elements or AREs at their 3' untranslated regions (UTR) [3,4]. For example, in yeast, AREs stimulated the shortening of poly adenine (poly A), and two kinds of degradation pathways followed. One is 5'-to-3' exonuclease access by removal of the 5' cap structure. The other is 3'-to-5' digestion by a complex of exonucleases called exosome [5,6]. Genes required for these steps have been identified in yeast and were found to be conserved among eukaryotes. Although the mechanisms of AREs enhanced mRNA degradation are unknown, several groups provided evidence that 3'-to-5' degradation by the exosome may be the major pathway of decay for at least some mammalian mRNAs, including ARE-containing mRNA sequences [7-9]. The length of AREs also affected the half-life of mRNA. The nonamer UUAUUUAUU is a typical ARE, and the simple repeats, (AUUU)_n_A motif, is the well-known pattern of AREs. It has been shown that the number of ARE motifs correlated with the turnover of ARE-mRNAs such as GM-CSF [10,11]. Because of this, AREs are usually classified according to the number of the repeats [12].

It is known that the stabilization factor, such as HuD, is able to bind to AREs [13] and most AREs seem to function as destablizing factors. The overall importance of AREs in biology is that they can make certain critical gene products unstable. They include proto-oncogenes such as c-fos [14], c-myb [15], c-myc [16], and Pim-1 [17]. Another class of ARE-associated genes are immune response genes such as interferon [15,18] and interleukin [15,19-21]. Growth factors, such as Gro-α [22] and the vascular endothelial factor [23] in humans, are also known to be associated with AREs.

AREs consist of a great number of thymine (or uracil) and a few adenines. Alu repeats can be a source of poly-T regions in mRNA. Therefore, there is a possible link between ARE and Alu repeats.

Alu repeats are sequences of approximately 300 nucleotides (nt) transcribed by RNA polymerase III. The Alu region is then reverse-transcribed and inserted into a new location in the genome [24]. It can reach a copy number in excess of 500,000 in the human genome [25]. Alu repeats were thought to be inserted very early in primate evolution, approximately 65 million years ago (mya). Alu amplification appears to have reached a maximum rate between 35 and 60 mya, and is currently amplifying at only 1% of the maximum rate [26]. Statistical analyses have identified key diagnostic nucleotide positions in Alu sequences that define 12 subfamilies. J class is the oldest one, S class is intermediate, and Y class is the newest. The majority of Alu retrotranspositions were completed at least 30 mya when the Alu-Sx subfamily, which accounts for half of all human Alu sequences, and the Alu-Sp and Alu-Sq subfamilies became unable to replicate [27-30]. Alu repeats account for 6–13% of the human genome [31] and were identified in 5% of 1,616 human full-length cDNA. Of the 5%, 82% were found in the 3' UTR, while 14% were located in the 5' UTR, and very rarely in the coding region [32]. The common role of Alu at 3' UTR has not been reported, although there is one specific case that the chemical, PMA, can bind to Alu at 3' UTR and increased mRNA half-life [43].

**Figure 1:**
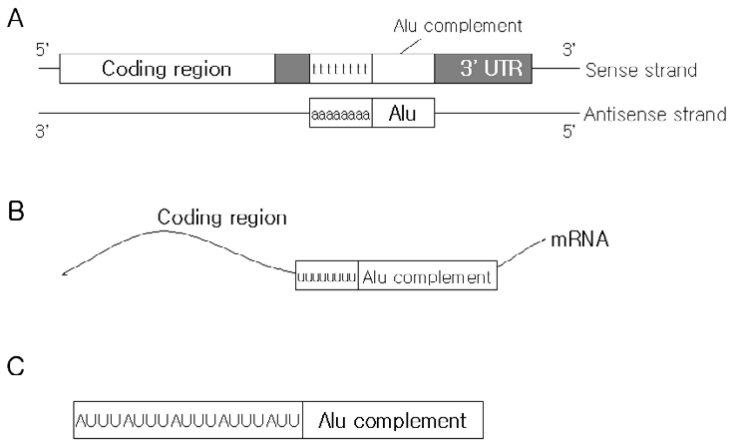
**The schematic diagram of poly-thymine (poly-T) generation by Alu.**(A) Alu contains poly-adenine (poly-A) region at the end. It is shown as 'aaaaaaaa'. The poly-A of Alu at anti-sense becomes poly-T (complement of poly-A) at sense strand on DNA. It is shown as 'tttttttt'. (B) mRNA now contains a poly-uracile (poly-U) region after the transcription of poly-T region. (C) AU-rich elements are found in this poly-U region in (B).

We investigated the link between Alu sequence and the potential AREs (that have not been experimentally verified but contain ARE sequence patterns), and suggest that the complementary poly-adenine regions of Alu is one of the sources of AREs at the 3' UTR of mRNA. Figure 1A shows that the poly-adenine regions of Alu contained in the anti-sense strand on DNA complemented the poly-thymine regions in the sense strand; therefore, the poly-thymine regions on DNA transcribed the poly-uracil regions on mRNA (Figure 1B). We propose a mechanism on how Alu has been converted to AREs gradually. When adenine was inserted at a regular interval in the poly-T(U) regions, it eventually led to the generation of potential AREs. It is not clear why such a regular insertion occurs, but the phenomenon has also been found in other ARE-like sequences. Figure 1C shows transcribed ARE on mRNA [33,34].

## Results

**Figure 2:**
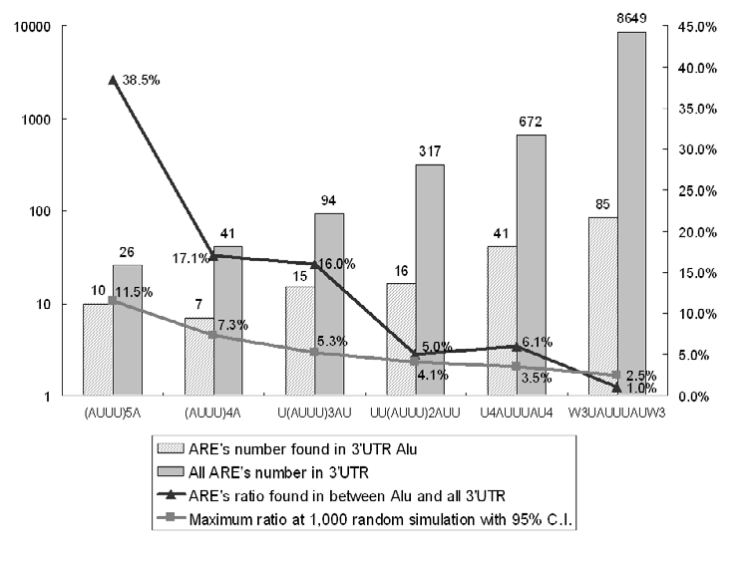
**ARE found in Alu at each class (Table 1).**The numbers of ARE found in all 3' UTR, the number of ARE found in the Alu sequence, the ratio between them, and the randomly simulated results among 1,000 times at each ARE class (Table 1). Only the maximum possible ratios of the randomly simulated range at 95% confidence interval (C.I.) were shown. X-axis is for ARE patterns in all the classes. The left Y-axis is for the number of AREs, and the right Y-axis is for the overlap ratios.

The results from the method are shown in Figure 2. In the ARE class I, marked as (AUUU)5A pattern in Table 1, 26 AREs were found in all 21,121 mRNA 3' UTR. 38.5% of 26 AREs included in the class I, were detected in Alu sequences at 3' UTR. When we did a simulation test for the 26 AREs and 1,504 Alu sequences by 1,000 times, with a 95% confidence interval (C.I.) threshold, it was statistically significant (see the statistical analysis of the search results in the Methods section). In other words, 38.5% occurrences were out of the likelihood for random overlaps of Alu and ARE patterns in the human genome. In the ARE class II (Table 1, (AUUU)4A pattern), 41 were found in all 3' UTR, and 7 were detected in Alu sequences among them (17.1%). The simulation results showed the 17.1% was less than the maximum random range of 7.3%. Therefore, class II data also showed a significance between ARE patterns and Alu. In class III (Table 1), 94 AREs were discovered from all 3' UTR. 15 out of 94 AREs were located in Alu sequences (16.0%). 16% was also statistically significant with the given sample size. In classes IV and V, 5% and 6.1% of ARE were found in Alu, respectively. These results were still out of the random chance distribution, although they were relatively less significant than the previous classes. In class VI, only 85 out of 8,649 AREs were detected in Alu (1%), and it is an insignificant hypothesis that the class VI pattern is associated with Alu sequences.

## Discussion

The possible mechanism of how AREs originated from Alu is as follows: Alu is a special sequence that contains a poly-adenine (poly-A) region at its end. The poly-A region plays an important role in the retroposition mechanism of Alu [35]. It is known that the products of LINE (L1) transposon bind the poly-A of Alu. This enables Alu to retroposition [36,37]. When Alu with poly-A are inserted as above, it is in the double helix form with the complementary poly-T. Therefore, the poly-T regions produce poly-uracil (poly-U) regions in mRNA when transcribed (Figure 1). We hypothesized that the poly-U regions generated from the Alu are the source of AREs after either random or directed mutation.

With this hypothesis, we suggest a new role for Alu was involved in the 3' UTR. It is well known that Alu affected gene expression at the 5' of genes and alternative splicing at the intron region [38,39]. However, no Alu role at the 3' UTR has been suggested yet. We could have applied the same test to Alu at 5' UTR region, but there were too few data sources [32].

## Conclusion

AREs are mediating sequences that affect the stabilization or degradation of biologically important genes' mRNA. However, their origin in evolution has not been clear. This report presents a hypothesis and statistical evidence that Alu was one of the sources of ARE generation or origin. A possible mechanism of ARE generation from Alu via retroposition and regular pattern mutation is suggested.

## Methods

### Human 3' UTR sequences

We used the RefSeq database from the National Center for Biotechnology Information (NCBI) for human 3' UTR sequences [41]. We extracted 3' UTR of CDS (coding sequence) from all the annotated mRNA sequences (mRNA_Prot, 2004.9.13). The number of 3' UTR was 21,121 and the average length was 996 bp. We used the Biojava package [42] to extract only 3' UTR with Genbank's feature information. The number of 3' UTR was 21,121 and the average length was 996 bp.

### Alu sequence and AU-rich element (ARE) pattern detection

AREs were searched for in the all 3' UTR (Table 1). An in-house java program was used to search for these AREs. While the number of AUUUA repeats decreased, the T flank region increased to 21 bp. Each ARE was allowed within one base mismatch. This is a stricter mismatch criterion than the one of AU-rich elements database (ARED) (the ARED trained experimental ARE data allow 10% of ARE length mismatch [24]). The RepeatMasker program was used for finding Alu. It is a program for finding repeat sequences [25]. After finding Alu sequences using RepeatMasker at 3'UTR, for each Alu, we recorded the position information (RefSeq ID, start and end position) for the next step analysis.

### Comparison between two search results

We compared the positions of 3' UTR Alu and ARE sequences. If an ARE was discovered within an Alu sequence, this ARE was regarded found in 3' UTR Alu. For example, if an Alu was found between 100–400 bp and an ARE was found between 99–129 bp, this ARE was in 3' UTR Alu in the same 3' UTR. If less than 50% of an ARE length was discovered in an Alu, we further check if there is 7 bp TSD (Target Site Duplication) between the Alu's end and the ARE's end [4]. For example, if an Alu is between 100–400 bp and an ARE between 80–110 bp, about 10 bp (33%) of the ARE belongs to the Alu. In this case, we check if there is 7 bp TSD between upstream region from 80 bp and downstream from 400 bp.

### Statistical analysis of the search results

To validate the significance of the searches, we calculated the random chance of the ARE and Alu sequence overlap at each class (Table 1).

#### Hypothesis

H0: ARE occurs in human 3'UTR independently from Alu.

#### Random sequence generation for statistical validation

The average length of 3' UTR of 21,121 human sequences was 996 bp. Within the long theoretical sequence of 21,121 × 996 bp, we generated 1,504 Alu (300 bp) and ARE sequences (21–13 bp). For example, 1,504 Alu and 26 (21 bp) AREs in ARE class I (Table 1) were generated following a uniform distribution as a control set. 1,504 and the number of AREs for ARE classes were the actual numbers of Alu and AREs found by our method. This random sequence generation was done 1,000 times with a 95% significance threshold.

#### Test results

In the ARE class I (Table 1), the significance range at a 5% error range was 0.0–11.5% (Figure 2) for the random chance of association between ARE patterns and Alu sequences. The results in other ARE classes are also shown in Figure 2. Our result of a 38.5% – 6.1% overlap between AREs and Alu, depending on ARE classes, was statistically significant. Therefore, hypothesis H0 was rejected.

## Authors' contributions

HJA conceived of this study, carried out the tests, and drafted the manuscript. JB participated in the design of the study and drafted the manuscript. KWL and DL amended and improved the design of the study. All authors read and approved the final manuscript.
